# Volcanic Ash Activates the NLRP3 Inflammasome in Murine and Human Macrophages

**DOI:** 10.3389/fimmu.2017.02000

**Published:** 2018-01-22

**Authors:** David E. Damby, Claire J. Horwell, Peter J. Baxter, Ulrich Kueppers, Max Schnurr, Donald B. Dingwell, Peter Duewell

**Affiliations:** ^1^Department of Earth and Environmental Sciences, Ludwig-Maximilians-Universität (LMU) München, Munich, Germany; ^2^Volcano Science Center, United States Geological Survey, Menlo Park, CA, Unites States; ^3^Department of Earth Sciences, Institute of Hazard, Risk and Resilience, Durham University, Durham, United Kingdom; ^4^Institute of Public Health, University of Cambridge, Cambridge, United Kingdom; ^5^Division of Clinical Pharmacology, Medizinische Klinik und Poliklinik IV, Klinikum der Universität München, Munich, Germany

**Keywords:** inflammasome, NLRP3, reactive oxygen species, lysosomal damage, volcanic ash, cristobalite, silica, mineral dust

## Abstract

Volcanic ash is a heterogeneous mineral dust that is typically composed of a mixture of amorphous (glass) and crystalline (mineral) fragments. It commonly contains an abundance of the crystalline silica (SiO_2_) polymorph cristobalite. Inhalation of crystalline silica can induce inflammation by stimulating the NLRP3 inflammasome, a cytosolic receptor complex that plays a critical role in driving inflammatory immune responses. Ingested material results in the assembly of NLRP3, ASC, and caspase-1 with subsequent secretion of the interleukin-1 family cytokine IL-1β. Previous toxicology work suggests that cristobalite-bearing volcanic ash is minimally reactive, calling into question the reactivity of volcanically derived crystalline silica, in general. In this study, we target the NLRP3 inflammasome as a crystalline silica responsive element to clarify volcanic cristobalite reactivity. We expose immortalized bone marrow-derived macrophages of genetically engineered mice and primary human peripheral blood mononuclear cells (PBMCs) to ash from the Soufrière Hills volcano as well as representative, pure-phase samples of its primary componentry (volcanic glass, feldspar, cristobalite) and measure NLRP3 inflammasome activation. We demonstrate that respirable Soufrière Hills volcanic ash induces the activation of caspase-1 with subsequent release of mature IL-1β in a NLRP3 inflammasome-dependent manner. Macrophages deficient in NLRP3 inflammasome components are incapable of secreting IL-1β in response to volcanic ash ingestion. Cellular uptake induces lysosomal destabilization involving cysteine proteases. Furthermore, the response involves activation of mitochondrial stress pathways leading to the generation of reactive oxygen species. Considering ash componentry, cristobalite is the most reactive pure-phase with other components inducing only low-level IL-1β secretion. Inflammasome activation mediated by inhaled ash and its potential relevance in chronic pulmonary disease was further evidenced in PBMC using the NLRP3 small-molecule inhibitor CP-456,773 (CRID3, MCC950). Our data indicate the functional activation of the NLRP3 inflammasome by volcanic ash in murine and human macrophages *in vitro*. Cristobalite is identified as the apparent driver, thereby contesting previous assertions that chemical and structural imperfections may be sufficient to abrogate the reactivity of volcanically derived cristobalite. This is a novel mechanism for the stimulation of a pro-inflammatory response by volcanic particulate and provides new insight regarding chronic exposure to environmentally occurring particles.

## Introduction

Explosive volcanic eruptions generate vast plumes of ash. Their fall-out can affect extensive populated areas beyond the immediate vicinity of a volcano. Ash is defined as the portion of the erupted ejecta less than 2 mm in diameter, and it is a heterogeneous mixture of glassy fragments, containing variable amounts of crystals and older rock from the volcanic edifice (lithics). Since the 1980 eruption of Mount St. Helens, USA, when ash impacted more than a million inhabitants in the Pacific Northwest, it has been known that a substantial fraction of the ejecta is of micron, or even sub-micron, size and, therefore, potentially capable of being a human respiratory health hazard (1).

An exacerbation of airway problems, such as asthma and chronic bronchitis, due to the heightened levels of fine particles in the ambient air suspended from the ash deposits, was an expected finding at Mount St. Helens (2). The presence of a significant amount of respirable crystalline silica, mainly as cristobalite, however, was wholly unforeseen and has led to repeated toxicological testing of the ash to help establish the implications of ash exposure for human health [see review in reference (3)]. In particular, there was concern regarding the risks of developing silicosis in the general population and outdoor workers due to the established consequences of crystalline silica exposure, mainly as quartz, in industrial settings (4). The eruptions of the Soufrière Hills volcano on Montserrat, West Indies, starting in 1995 and lasting over 15 years, led to similar intensive study of the volcanic cristobalite hazard (3, 5). Ongoing work has constrained the presence of cristobalite in ash to eruptions that involve lava domes or incorporate pre-existing, altered flow-units (5, 6). This is because cristobalite forms by secondary mineralization or hydrothermal alteration in these environments and, therefore, is not present in primary magmatic ejecta. These discoveries have defined the environmental side of the hazard; however, the capacity of volcanic cristobalite to incite disease remains enigmatic (7, 8).

Previously, we have observed no systematic difference in reactivity when comparing ash containing crystalline silica, predominantly as cristobalite, and ash containing negligible amounts of crystalline silica (9, 10). Experimentally, cristobalite-bearing volcanic ash has incited granuloma formation *in vivo* (1, 11), but it is consistently less inflammatory and fibrogenic than would be expected for a crystalline silica-bearing dust (3, 12). However, we have recently reported on the propensity of volcanic ash to initiate an inflammatory immune response *in vitro* in macrophages (10). Crystalline silica in other mixed-mineral dusts is known to be variably reactive (13), whereby its pathogenicity may be altered by inherent structural and chemical defects along with effects imparted by other constituents in a mixed-phase dust; indeed, structural and chemical defects of volcanic cristobalite (which contains up to 3 wt.% aluminum) together with its presence in a heterogeneous dust have all been previously implicated in its reduced potency (8, 14, 15). The conflicting results to date have hindered efforts to provide health risk assessments (16), which consider all available evidence from *in vitro* and *in vivo* toxicological tests. A mechanistic understanding of the hazard posed by volcanic ash is needed to resolve the existing conundrum and provide appropriate public health advice during future volcanic eruptions.

Inflammation plays a pivotal role in crystal-driven disease progression and can be observed in patients with particle-induced lung diseases (17). Although the exact mechanisms of how pathogenic particles drive inflammation is not completely understood, it has been shown that danger-associated molecular patterns, such as endogenous uric acid (gout) or cholesterol (atherosclerosis) as well as exogenous particles like asbestos (asbestosis) or crystalline silica (silicosis), share features of crystalline particles that activate the NLRP3 inflammasome (also known as CIAS1, NALP3, or cryopyrin) (18–20). The NLRP3 inflammasome belongs to the Nod-like receptor pyrin-containing family of cytosolic receptors and, together with the adapter molecule apoptosis-associated speck-like protein containing a CARD domain (ASC), it forms a multi-protein platform that recruits and activates caspase-1. Caspase-1 belongs to the inflammatory caspases and leads to the processing and secretion of the pro-inflammatory cytokines interleukin-1 beta (IL-1β) and IL-18 into their active forms (21). While the exact upstream mechanism of NLRP3 activation remains unclear and is part of ongoing studies, the current understanding of NLRP3 inflammasome assembly mainly consists of a two-hit mechanism. NLRP3, as well as the pro-form of IL-1β, is not constitutively expressed and needs transcriptional priming. The first step in activation can be achieved by the germ line-encoded TLRs that usually sense microbial cell wall components or viral DNA and RNA molecules. The NLRP3 inflammasome senses crystalline danger signals that can occur during autoinflammatory diseases, such as gout or atherosclerosis, and environmental diseases, such as silicosis or asbestosis (18, 20, 22). IL-1 cytokines are potent mediators of innate immunity in response to crystalline silica exposure (23, 24) and have been implicated in the pathophysiology of human and experimental diseases (25, 26).

Here, we report on the propensity of volcanic cristobalite to activate the NLRP3 inflammasome, in the wake of a series of in-conclusive toxicological investigations of ash from recent major eruptions. The NLRP3 inflammasome has emerged as a central mechanism in mediating cellular responses to various endo- and exogenous signals and particles related to environmental and life-style diseases. Given the established hazard posed by respirable crystalline silica in occupational settings, the capacity of volcanic ash to stimulate IL-1β release by macrophages *in vitro* (10), and the observation that instigation of chronic disease by crystalline silica is NLRP3-dependent (27, 28), we have chosen to test the ability of cristobalite-bearing volcanic ash to promote inflammation by activating the inflammasome pathway.

## Materials and Methods

### Volcanic Ash Sample and Major Component Control Particles

Ash sample MRA5/6/99 is a respirable sample isolated from fresh ash that fell at Soufrière Hills volcano, Montserrat, on 5 June 1999. The ash was generated during a dome-collapse event, a particular style of eruption known to produce fine-grained, cristobalite-rich ash (5, 29). The respirable fraction was isolated using the Minisplit classification system at 16,000 rpm (British Rema, Sheffield, UK), which segregates particles within a vortex. The bulk tephra (sieved to 1 mm) was first separated to give a sub-10 μm fraction, and this fraction was further separated to give the sub-4 μm fraction. Sample MRA5/6/99 has been used extensively in ash characterization and toxicity studies; the ash is characterized in detail by Horwell et al. (29) and the crystallographic properties of the cristobalite it contains by Damby et al. (14). The sample comprises ~15 wt.% crystalline silica as cristobalite, with the other major constituents being volcanic glass (amorphous silica) and plagioclase feldspar; additional minor phases identified were hornblende, orthopyroxene, titanomagnetite, and oxides (10, 29).

Pure-phase mineral samples of the primary components were analyzed alongside MRA5/6/99 to constrain their reactivity in the NLRP3 inflammasome model. Cristobalite was synthesized by heating ultra-high purity quartz glass (Heraeus HOMOSIL^®^ 101, Hanau, Germany) for 12 h in a platinum crucible at 1,600°C in air. As a representative feldspar, we sourced labradorite (feldspar), an intermediate member of the plagioclase series, from the Bavarian State Collection for Mineralogy (Munich, Germany). Anhydrous andesite glass was produced from high temperature (1,450°C) melting of a sub-sample of Soufrière Hills pumice (described below) in a Nabertherm HI 04/17 furnace (Lilienthal, Germany) in air for 12 h. The sample was then stirred under similar conditions in a second furnace to ensure a homogenous melt and rapidly quenched to produce glass. A cristobalite-free pumice sample from the 12 July 2003 eruption of Soufrière Hills volcano was included as the mineralogy is similar to MRA5/6/99, and it thus serves as a natural material control for the minor phases identified above [see reference (30)]. All componentry samples were ground dry in a mortar and pestle prior to use.

### Sample Characterization

The particle size distributions of the samples were measured using a Coulter LS 230 Analyzer (Beckman Coulter Inc., CA, USA). Data were collected using the following refractive indices: 1.63 for Soufrière Hills ash, as optimized in Horwell (31), 1.49 for crystalline silica, 1.56 for feldspar, and 1.53 for synthetic andesitic glass (32). Data are the average of three 60-s runs and are analyzed according to the Mie scattering theory. The surface area of sample MRA5/6/99 is 3.6 m^2^/g as measured by the BET method of nitrogen adsorption (33).

Imaging of the volcanic ash sample was carried out on a Hitachi SU-70 FE-SEM (Hitachi, Ltd., Tokyo, Japan) in the GJ Russell Microscopy Facility, Department of Physics, Durham University. Images were collected at an operating voltage of 6.0 kV and a working distance of 14 mm. Sample mineralogy was confirmed by powder X-ray diffraction on a Bruker AXS D8 ADVANCE (Bruker Corp., MA, USA) with DAVINCI design in 2θ reflection mode using Cu radiation and a Ni filter in the Department of Chemistry, Durham University.

### Cell Lines and Reagents

Wild-type and knock-out bone marrow-derived immortalized mouse macrophage cell lines (iMΦ) were generated with a recombinant retrovirus, carrying v-myc and v-raf(mil) oncogenes, as previously described by Hornung et al. (22). Cells were cultured in DMEM supplemented with l-glutamine, 10% FCS (all Gibco, Darmstadt, Germany) and ciprofloxacin (Sigma, Taufkirchen, Germany). Freshly isolated human peripheral blood mononuclear cells (PBMCs) from randomly selected donors were obtained using density gradient centrifugation with subsequent red blood cell lysis. Cells were kept and stimulated in RPMI supplemented with l-glutamine, 10% FCS (all Gibco, Darmstadt, Germany) and ciprofloxacin (Sigma, Taufkirchen, Germany). iMΦ were seeded at a density of 1 × 10^5^ cells/96 well and PBMC at a density of 1 × 10^6^ cells/96 well and primed with 200 ng/ml (iMΦ) or 100 pg/ml (PBMC) lipopolysaccharide (LPS, InvivoGen, Toulouse, France) for 2 h. For inhibitor studies, latrunculin A (Lat A), CA-074-ME, (2R,4R)-4-aminopyrrolidine-2,4-dicarboxylic acid (APDC), and CP-456,773 (CRID3, MCC950) were used at 20 µM and applied 1 h prior to stimulation. Cells were then stimulated with ash and componentry samples as indicated or with 5 mM adenosine triphosphate (ATP), 200 ng/ml polydeoxyadenylic acid • polythymidylic acid (dAdT), 10 µM nigericin (all Sigma Aldrich, Taufkirchen, Germany), or 1 mM H-Leu-Leu-OMe Hydrochloride (Santa Cruz, Heidelberg, Germany). After 6 h, IL-1α, IL-1β, IL-6, and TNFα cytokine levels in supernatants were measured by ELISA (all BD Biosciences, Heidelberg, Germany) or for supernatants and cell lysates with western blot analysis.

### Western Blot

Immortalized macrophages and human PBMC were seeded and stimulated, as described above, but in serum-free media. Supernatants were removed and processed for protein precipitation. Briefly, equivalent amounts of methanol and 20 vol.% chloroform were added to supernatants, vortexed, and centrifuged at 12k rcf for 5 min. The top aqueous layer was discarded and methanol was added to remove remaining chloroform from precipitated whole-protein pellets. Samples were centrifuged at 12k rcf for 5 min, supernatants were carefully removed and the protein pellets were air-dried. Precipitates were resolubilized in Lämmli buffer and heated at 95°C for 5 min. Samples were separated using SDS-PAGE and blotted for protein detection. Membranes were blocked with 3% BSA and incubated with goat anti-mouse caspase-1 p20 (Santa Cruz, Heidelberg, Germany), goat anti-mouse IL-1β, or goat anti-human IL-1β (all R&D Systems, Wiesbaden-Nordenstadt, Germany) pAb overnight. HRP-coupled donkey anti-goat IgG secondary antibodies were incubated for 2 h. HRP-coupled β-actin IgG mAb served as loading control.

### Light Microscopy

iMΦ were treated with 500 µg/ml volcanic ash for 6 h. Particle-treated cells were imaged by light microscopy using a Zeiss Axiovert 200 M microscope (Zeiss, Oberkochen, Germany). Cell images were acquired using a 20× objective.

### Confocal Laser Reflection and Immune Fluorescence Imaging

iMΦ were seeded in glass-bottom dishes (Thermo Scientific, Darmstadt, Germany) at a density of 1 × 10^5^ cells/ml in complete DMEM and allowed to adhere. Cells were incubated with the quenching dye conjugate DQ-Ovalbumin (DQ-OVA) in the presence or absence of MRA5/6/99 for 4 h. Cells were washed and counterstained with the membrane dye Alexa Fluor^®^ 647-conjugated cholera toxin B subunit (Ctx B) and the nucleic acid stain Hoechst 33342 (Invitrogen, Karlsruhe, Germany). Combined reflection and immune fluorescence data were acquired using a Leica TCS SP5 AOBS confocal laser scanning microscope with 63× magnification (Wetzlar, Germany).

### Statistical Analysis

All results are expressed as mean ± SD. Comparisons and significance between two groups was assessed by Student’s *t*-test. The level of significance is assigned to *p* ≤ 0.05.

## Results

### Processing of Volcanic Ash by Macrophages

**Figure 1:**
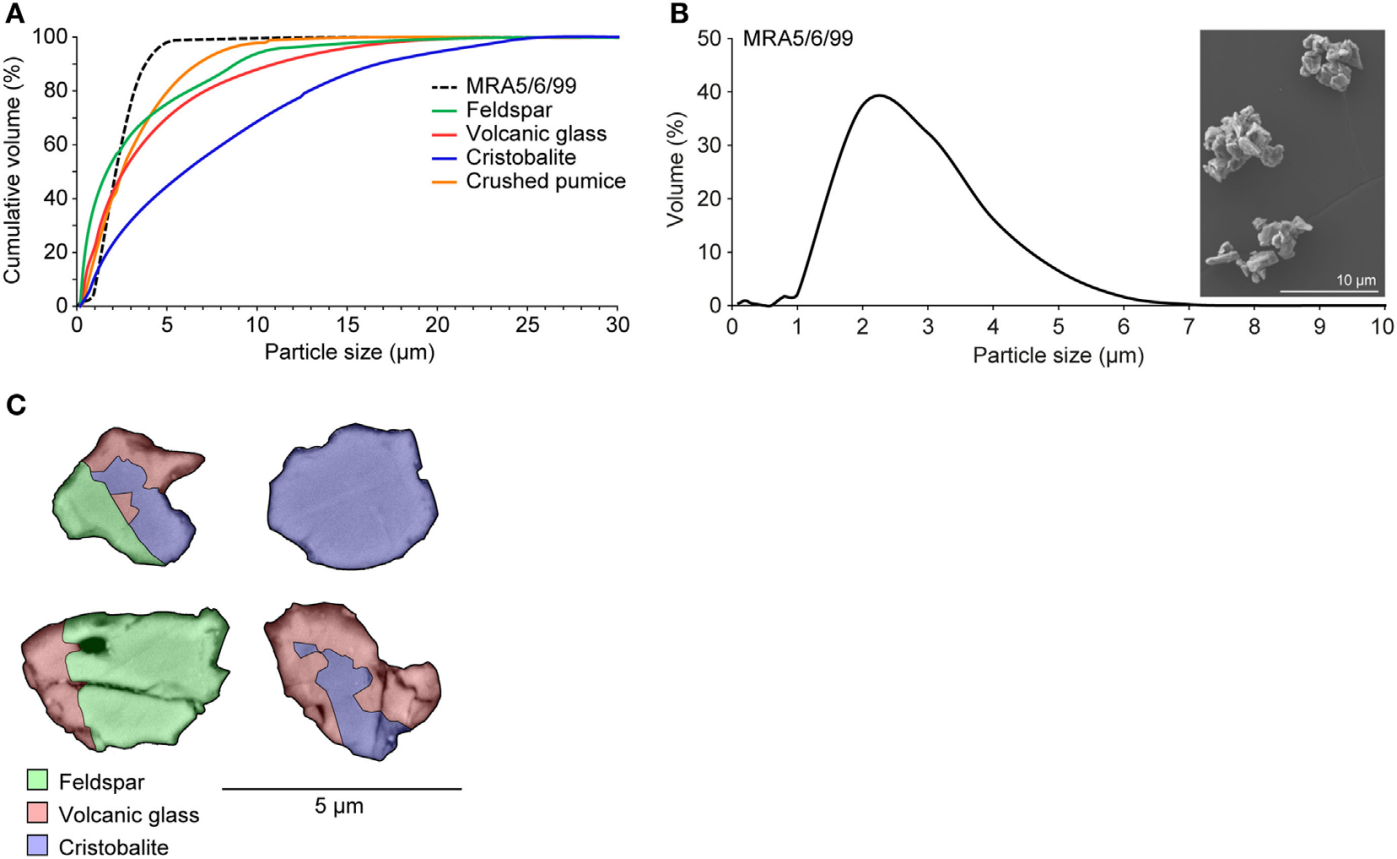
Characteristics of isolated volcanic ash and pure-phase componentry. **(A)** Cumulative particle size distributions of volcanic ash sample MRA5/6/99 and componentry samples. **(B)** Particle size distribution and SEM image of volcanic ash sample MRA5/6/99 (×4.00k magnification). All particle size data are the average of three runs. **(C)** False-color backscatter SEM image of ash in cross section (×10k magnification) evidencing the heterogeneous distribution of predominant phases: cristobalite, feldspar, and volcanic glass. Electron dispersive X-ray spectroscopy was employed for mineral identification. Images were collected at 8.0 kV and a 14.5 mm working distance.

Macrophages are a first line of defense against inhaled particles and are responsible for coordinating an inflammatory immune response. Therefore, they are key targets for *in vitro* assessment of the hazard posed by atmospheric particles. Diverse crystalline material has been reported to activate the NLRP3 inflammasome *via* phagosomal destabilization with lysosomal content leaking into the cytosol. Consequently, this leads to activation of a plethora of endoproteases and oxidative stress molecules by a not yet completely understood mechanism (22, 34). However, particle size is known to be important for entering the endosomal compartment, as reported for silica crystals with an average size of 1–2 µm (22). Volcanic ash comprises a wide range of particle sizes depending on the fragmentation efficiency of the eruption (35), but can contain a substantial respirable component (31). To represent pulmonary exposures, we used an isolated respirable ash sample, obtained through fractioning and sieving methods previously described (29), that derived from a dome-collapse event at Soufrière Hills volcano (MRA5/6/99) and synthetic particles of its corresponding componentry (Figure 1A). The ash sample is particularly fine-grained: scanning electron microscopy and particle sizing data of the isolated sample show that the particles are <5 µm, with a mode of 2 µm (Figure 1B). Backscatter SEM imaging reveals a heterogeneous distribution of volcanic glass, feldspar, and cristobalite as the predominant phases (Figure 1C). The ash sample has been previously reported to be successfully internalized by differentiated THP-1 cells, the human monocytic cell line (10).

### Volcanic Ash Induces Inflammation and Leads to IL-1β Secretion

**Figure 2:**
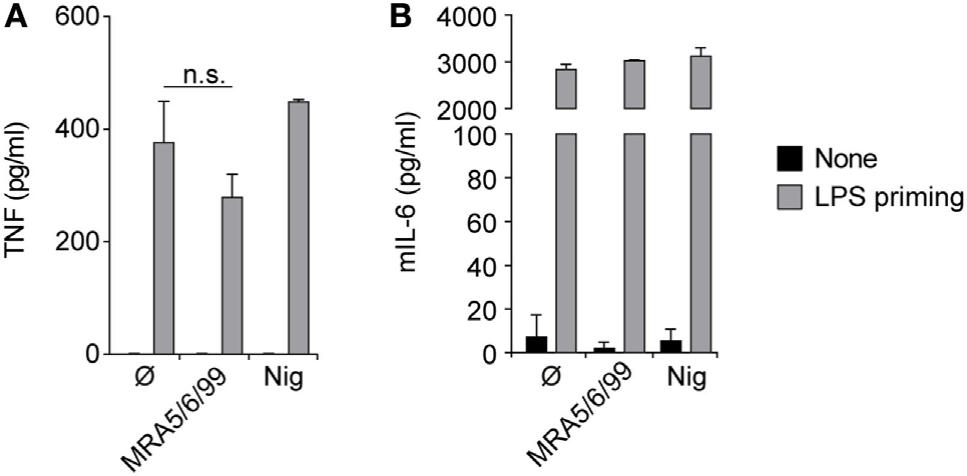
Volcanic ash does not induce a primary inflammatory response. Lipopolysaccharide (LPS)-primed and unprimed wild-type murine macrophages were stimulated with volcanic ash sample MRA5/6/99 or nigericin and **(A)** TNFα and **(B)** IL-6 from supernatants were examined by ELISA. Representative data from two independent experiments are shown.

**Figure 3:**
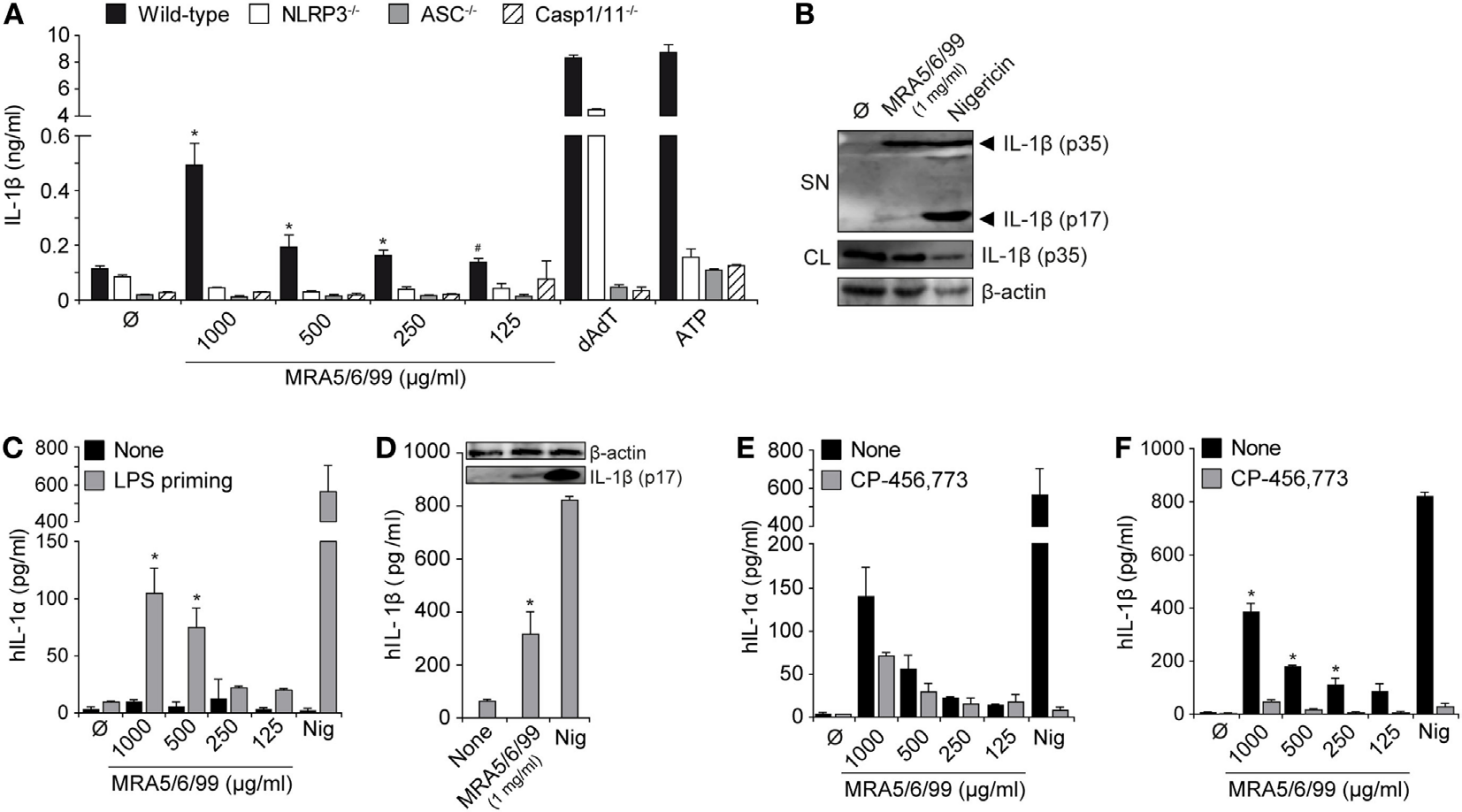
Volcanic ash-induced IL-1β production is NLRP3 inflammasome dependent. Lipopolysaccharide (LPS)-primed macrophages from wild-type or knock-out mice (NLRP3^−/−^, ASC^−/−^, and Casp-1/11^−/−^) were stimulated with MRA5/6/99 at indicated concentrations. **(A)** IL-1β from supernatants (SN) was measured with ELISA. **(B)** Mature IL-1β (p17) inSN and pro-IL-1β (p35) from cell lysates (CL) of cells stimulated with 1 mg/ml MRA5/6/99 was assessed by western blot. β-actin IgG mAb served as loading control. **(C,D)** Primary human peripheral blood mononuclear cells (PBMC) were either LPS-primed or left untreated and subsequently stimulated with MRA5/6/99. **(C)** IL-1α and **(D)** IL-1β levels in SN were determined by ELISA. Insertion **(D)** shows the western blot of bioactive IL-1β (p17) detected from SN. **(E,F)** IL-1α and IL-1β in SN of ash-treated human PBMC in the presence or absence of the NLRP3 inhibitor CP-456,773, assessed by ELISA. Positive controls are poly(dAdT), as a NLRP3-independent inducer, and adenosine triphosphate (ATP) or nigericin, as NLRP3-dependent inducers of IL-1β. Representative data from two independent experiments performed as triplicates are shown. **(A)** *p ≤ 0.05, compared to NLRP3^−/−^, ASC^−/−^, Caspase-1^−/−^; ^#^*p* ≤ 0.05, compared to NLRP3^−/−^ and ASC^−/−^; **(C,D)** *p ≤ 0.05, compared to none (Ø); **(E,F)** *p ≤ 0.05, compared to inhibitor (CP-456,773).

The inhalation and deposition of particulate pollutants, such as diesel exhaust, asbestos, or silica, in the small airways is known to induce inflammatory responses leading to sustained inflammation, lung fibrosis, and cancer (22, 25). The abundance of cristobalite in ash from Soufrière Hills volcano, as well as in ash from a large number of other dome-forming volcanoes [see (7) and references therein], and the potential for ash to induce the secretion of inflammatory cytokines prompted us to test the inflammatory response to ash particles further. Accordingly, we treated mouse macrophages with ash sample MRA5/6/99 and determined levels of TNFα, IL-6 and IL-1β in culture supernatants (Figures 2 and 3A). As expected, cells responded normally to the microbial TLR4 agonist LPS with high levels of TNFα and IL-6; however, no relevant cytokine secretion was detectable after exposure to volcanic ash without prior LPS stimulation (Figures 2 and 3A). Compared to other pro-inflammatory cytokines, pro-IL-1β lacks a signaling peptide sequence to exit the cell *via* golgi translocation. IL-1β is not constitutively expressed and requires transcriptional upregulation in response to a canonical NF-κB pathway or microbial stimuli, such as LPS, sensed by TLR receptors. As IL-1β itself is predominantly activated in a NLRP3/caspase-1-dependent manner, we investigated the involvement of the NLRP3 inflammasome in the response to volcanic ash.

### Volcanic Ash-Induced IL-1β Secretion Involves the NLRP3 Inflammasome and Caspase-1 Activation

Activation of the NLRP3 inflammasome results in assembly with the adaptor protein apoptosis-associated speck-like protein containing a CARD (ASC) and subsequent activation of caspase-1, with the release of active IL-1β. We stimulated immortalized wild-type macrophages or cells with deficiencies in the inflammasome signaling components NLRP3, ASC, or Caspase-1/11 with LPS (TLR4 ligand) and subsequently incubated the cells with volcanic ash sample MRA5/6/99 (Figure 3A). Only LPS-primed macrophages responded with high amounts of IL-1β, pointing toward the involvement of the NLRP3 inflammasome. Analysis of supernatants revealed a strong and dose-dependent secretion of IL-1β from wild-type macrophages whereas cells with defects in NLRP3 signaling (NLRP3^−/−^, ASC^−/−^, and Caspase-1/11^−/−^) components failed to secrete IL-1β in response to volcanic ash exposure (Figure 3A). As control served the double-stranded DNA mimic dAdT that signals NLRP3 independently *via* the DNA sensor absent in melanoma 2 (AIM2), still requiring the adapter molecule ASC as well as caspase-1 for the processing of IL-1β (36). ATP signaling *via* the ligand-gated cation channel P2×7 served as the NLRP3-dependent positive control. To further confirm proteolytic cleavage of IL-1β into its active form (p17 subunit), we performed a western blot analysis of whole cell lysates and the corresponding supernatants. As expected, volcanic ash was able to induce activation of mature IL-1β, however, in a moderate way compared to the positive control nigericin, a highly potent pore-forming toxin (Figure 3B).

We further analyzed the ash-induced release of IL-1 family cytokines (IL-1α and IL-1β) from freshly isolated primary human PBMCs (Figures 3C–E). The closely related homolog IL-1α is, in contrast to IL-1β, biologically active, although little is known about its activation and secretion. IL-1α represents a key alarmin from dying cells and plays a pivotal role in acute particle-induced lung inflammation (37). Here, we show that volcanic ash samples alone are not capable of inducing IL-1 secretion in the absence of LPS priming. In contrast, LPS-activated PBMCs secreted high amounts of IL-1α and IL-1β in a dose-dependent manner (Figure 3C). Secretion of bioactive IL-1β again was assessed by western blot showing the cleaved p17 fragment in supernatants of ash- or nigericin-treated samples (Figure 3D). Finally, the contribution of the NLRP3 inflammasome was confirmed using the specific NLRP3 inflammasome inhibitor CP-456,773 (CRID3, MCC950) that has been described earlier (38). The data show that CP-456,773 potently inhibited the release of IL-1β and, to a lesser extent, IL-1α (Figures 3E,F). Despite the well-described functions of IL-1β, IL-1α exists in two different forms, surface-bound pro-IL-1α and a mature secreted form. The fact that IL-1α can, on one hand, signal independently of inflammasome activation but, on the other hand, requires caspase-1 and mature IL-1β for secretion suggests the involvement of other inflammatory pathways, which are yet to be exactly investigated (39).

### Cristobalite Is the Apparent Driver of IL-1β Secretion by Volcanic Ash

Volcanic ash is a heterogeneous dust, the componentry of which can vary substantially amongst eruptions and even within the same eruption. Therefore, identifying the phase(s) responsible for the observed reactivity is critical for hazard assessment in the event of an eruption, where ash componentry analysis is a first priority in rapid-response efforts (9, 40). Despite the fact that all of the mineral phases present in volcanic ash are not bio-soluble on the timescale of our experiments, or on the expected timescale of phagocytosis *in vivo*, physiologically relevant cations (K^+^, Na^+^, Ca^2+^) could be leached from the glass component *via* intra-cellular processing of particles in a matter of hours to days (41) by lysosomal fluid, a buffered, acidic (pH 5.5) solution largely comprising citric acid (42). This may augment the intra- and extra-cellular cation budget, an established condition implicated in inflammasome assembly and activation (43, 44). As neither the compositionally equivalent glass sample nor pumice, which is predominantly composed of glass, were comparably reactive, we discount particle alteration and glass leaching as primary controls on NLRP3 and subsequent caspase-1 activation by volcanic ash. This conclusion is substantiated by minimal (and equivalent) extraction of K^+^ and Na^+^ in leaching experiments conducted in dilute citric acid, the primary organic ligand in lysosomal fluid, and at a relevant pH (41). Therefore, we do not expect any significant alteration to the bulk phase assemblage throughout the experimental exposures, and contend that single-phase exposures are appropriate to probe the reactivity of the mixed-phase volcanic ash.

**Figure 4:**
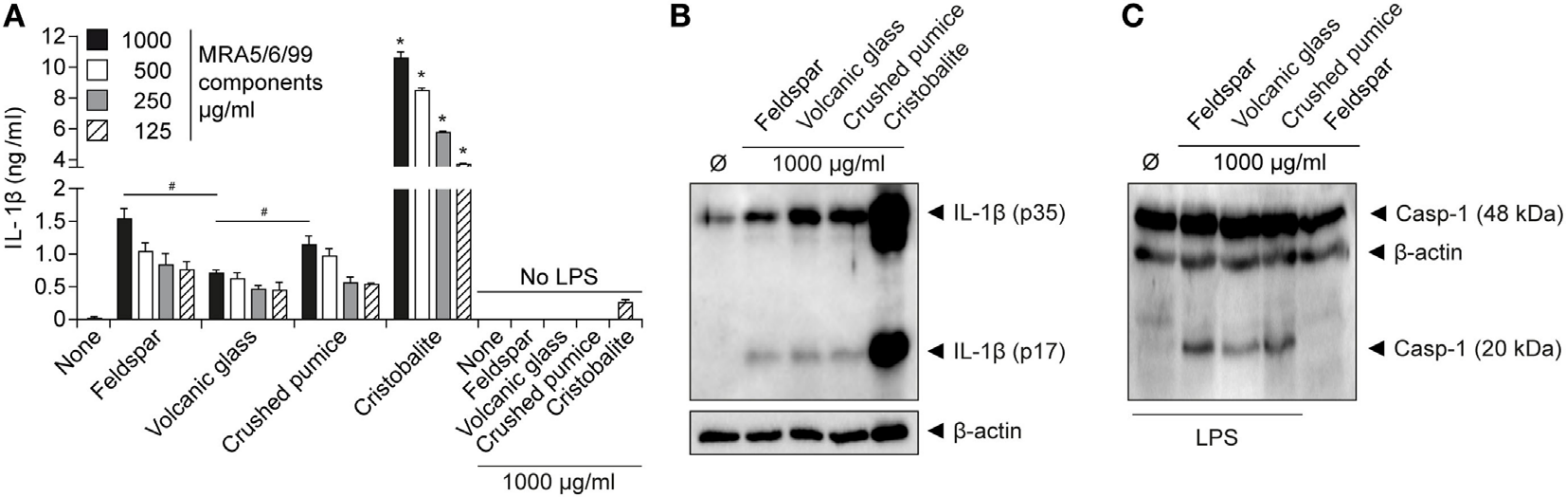
Cristobalite is the main component for volcanic ash-induced IL-1β release. Lipopolysaccharide (LPS)-primed and unprimed wild-type murine macrophages were stimulated with the major ash components (feldspar, volcanic glass, and cristobalite) and natural crushed pumice sample at indicated concentrations. **(A)** IL-1β from supernatants (SN) was examined by ELISA and **(B)** assessment of mature IL-1β (p17) from SN of LPS-primed macrophages by western blot. **(C)** Activated caspase-1 (p20 subunit) in supernatant detected by western blot analysis. Representative data from two independent experiments performed as triplicates are shown. *p ≤ 0.05, compared to other components; ^#^*p* ≤ 0.05.

Of the componentry samples, the silica compound cristobalite triggered the strongest dose-dependent release of IL-1β (Figure 4A), suggesting it is the predominant driver behind production of IL-1β by volcanic ash. This aligns with the established propensity of cristobalite to induce production of IL-1β through activation of the NLRP3 inflammasome (45). We note, however, that intermediate levels of IL-1β were detected as well as the active caspase-1 subunit p20 in response to treatment of LPS-primed macrophages by all componentry samples (Figures 4B,C), thereby strengthening the evidence of NLRP3 involvement.

### Volcanic Ash Acts through Translocation Into the Cytosol *via* Lysosomal Disruption and Reactive Oxygen Species (ROS) Production

**Figure 5:**
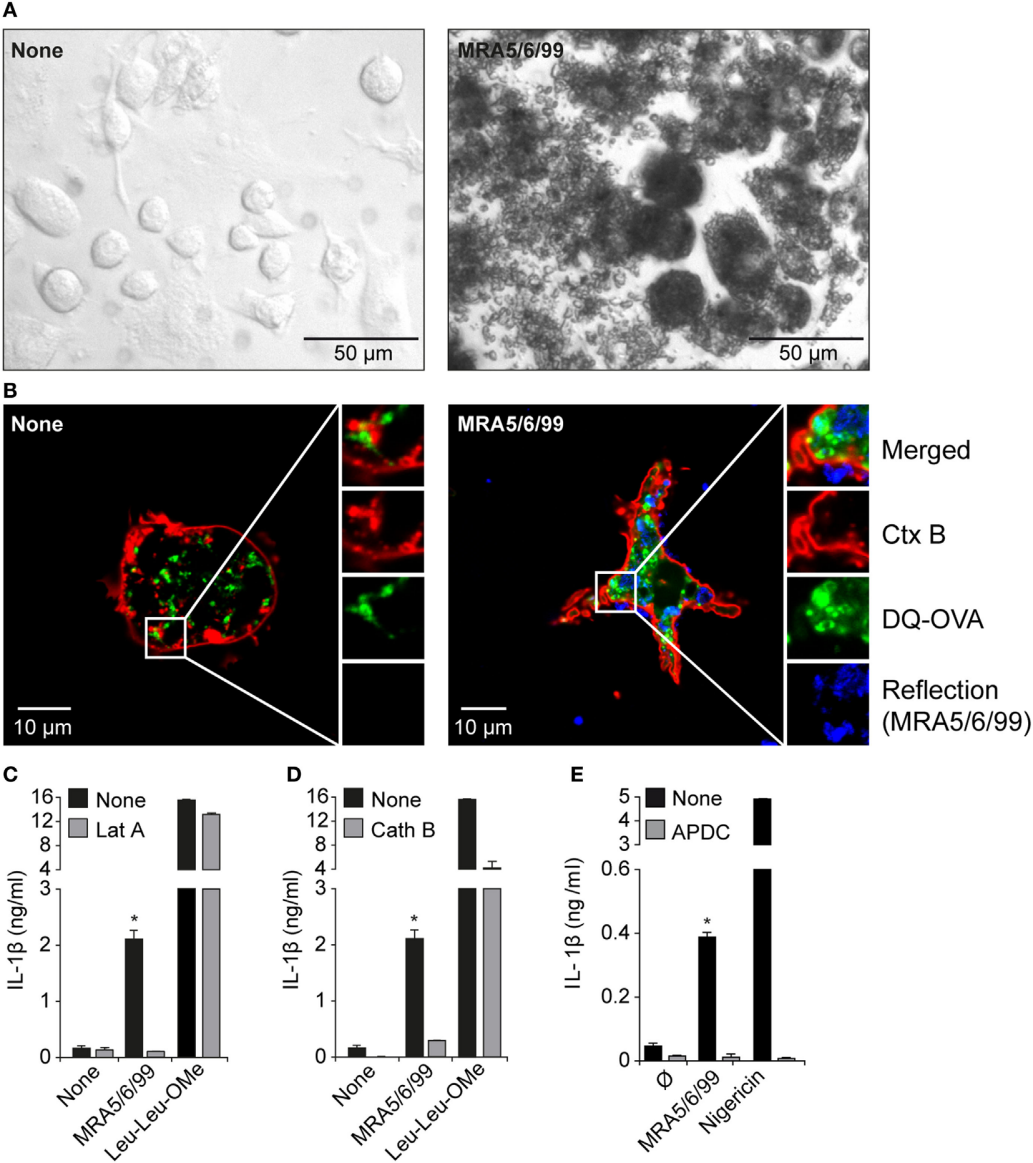
Internalization of volcanic ash by macrophages induces lysosomal damage and reactive oxygen species (ROS). **(A)** Light microscopy (20× magnification) and **(B)** laser scanning microscopy images (63× magnification) of untreated wild-type murine macrophages and macrophages treated with volcanic ash sample MRA5/6/99 for 4 h and stained with Ctx B (red) and DQ-Ovalbumin (DQ-OVA) (green). **(C–E)** LPS-primed immortalized macrophages were stimulated with 1 mg/ml MRA5/6/99 in the presence or absence of the endocytosis inhibitor latrunculin A (Lat A) **(C)**, the cathepsin B inhibitor CA-074-Me **(D)**, or the ROS scavenger (2R,4R)-4-aminopyrrolidine-2,4-dicarboxylic acid (APDC) **(E)**. Secretion of IL-1β in supernatants was measured by ELISA. Leu-Leu-OMe and nigericin served as lysosomotropic positive control and NLRP3-dependent inflammasome activator, respectively. Representative data from two independent experiments are shown. *p ≤ 0.05, compared to inhibitors (Lat A, Cath B, APDC).

To demonstrate that volcanic ash was internalized by macrophages, we incubated immortalized macrophages with the isolated ash sample for 4 h and imaged untreated and particle-treated cells by light microscopy (Figure 5A). The material was taken up by the cells, resulting in massive clustering of particles within the cell, and the intensity of optical refraction (darker cells) illustrates the capacity of the macrophages in particle clearance. This aligns with other recent observations of successful phagocytosis of volcanic ash by macrophages with little associated decrease in cell viability (10, 46). We next combined confocal laser reflection and fluorescence imaging to further consider cellular uptake of volcanic ash. We co-incubated the cells with the quenching dye DQ-OVA that, upon proteolytic processing, allowed us to monitor the endo-lysosomal compartment of living cells. As expected, DQ-OVA showed a distinct vesicular distribution in the absence of ash. In contrast, ash-treated cells showed enlarged vesicles with enhanced particle uptake (Figure 5B). Furthermore, ash and DQ-OVA translocated to the cytosol and showed partial co-localization. Faint fluorescence across the entire cytosol is evident of spreading and advanced dilution of the DQ quenching dye.

To test if endosomal uptake was necessary for ash-induced cytokine production, we incubated cells with the endocytosis inhibitor Lat A prior to ash exposure of the cells and measured IL-1β secretion (Figure 5C). Lat A completely inhibited ash-induced IL-1β release, whereas it had no influence on cytokine secretion mediated by the pore-forming toxin nigericin, strengthening the role of lysosomal uptake in response to volcanic ash. The data further reveal that lysosomal uptake seems to be the main uptake mechanism leading to lysosomal rupture with subsequent IL-1β release. In terms of lysosomal destabilization, enzymes become proteolytically active and are released into cytosol for inflammasome activation. Cathepsins are protein-degrading enzymes that become cleaved upon lysosomal maturation and are released as active forms into the cytosol. The cathepsin family comprises multiple proteins involved in IL-1β processing with cathepsin B being a well-described player involved in NLRP3 activation (22, 47). To test the contribution of cathepsins in volcanic ash-induced NLRP3 activation, we pre-incubated wild-type macrophages with the cathepsin B inhibitor CA-074-Me prior to ash exposure and measured IL-1β secretion (Figure 5D). Inhibition of cathepsin B completely inhibited IL-1β secretion, compared to partial effects observed with the lysosomotropic agent l-leucyl-l-leucine methyl ester (Leu-Leu-OMe). With respect to particulate and non-particulate structures, the data further show that, in terms of NLRP3-dependent IL-1β processing, redundant roles of cathepsin family members might play a role, as previously described (48).

Particulate substances or fibers are likely candidates for the initiation of ROS (19). While ROS are an important feature of maintaining immune homeostasis, excessive production results in inflammation and carcinogenesis as postulated for inhaled asbestos fibers, with mechanisms such as “frustrated” phagocytosis being discussed (19, 49, 50). It has been shown that, in most scenarios, ROS are associate stimulators of particle-induced NLRP3 inflammasome activation. We show here that ROS production is involved in ash-induced IL-1β release, using the small-molecule ROS scavenger (2R,4R)-4-aminopyrrolidine-2,4-dicarboxylic acid (APDC), which completely abrogated IL-1β secretion (Figure 5E). Previous work has shown that volcanic ash has the capacity to generate large amounts of ROS, largely due to the presence of non-crystalline-silica minerals (33, 51); however, the primary non-crystalline-silica components of volcanic ash have no history of inducing an inflammatory response (11, 52). In the present study, we observed low but dose-dependent production of IL-1β by the major constituents but, critically, the crushed pumice sample, which contains a similar assemblage of “non-crystalline-silica minerals,” was minimally reactive. Therefore, while auxiliary mineral-based ROS generation may be partially implicated in the observed volcanic ash reactivity, volcanic cristobalite is the likely candidate for NLRP3-mediated IL-1β production by volcanic ash.

## Discussion

There is mounting evidence that, while not overtly toxic, volcanic ash exposure can result in general insult with the potential for chronic toxicity by inciting a low, but significant, pro-inflammatory response and resulting in delayed inflammation *in vivo* (10, 11). In this study, we identify a prominent role for ash-induced inflammation, and provide the first mechanism-specific response of macrophages to volcanic ash, whereby phagosomal uptake results in significant secretion of mature IL-1β. This secretion is caspase-1 and ASC dependent, identifying the involvement of an inflammasome-mediated pathway. Critically, silencing of the murine NLRP3 gene as well as inhibition of human NLRP3 completely abolished ash-induced IL-1β secretion. We show that endocytosis and cathepsins are involved in the induction of IL-1β secretion, which indicates that lysosomal maturation, with subsequent rupture and loss of lysosomal contents into the cytosol, is one major mechanism of volcanic ash-induced inflammation *in vitro*. The NLRP3 inflammasome as an ash-responsive element appears to be predominantly mediated by the crystalline silica phase (cristobalite), although other phases induced a low-level response. The componentry of volcanic ash is variable and intrinsically linked to eruption history, and, by isolating the reactivity of cristobalite, these data provide a new avenue for considering the propensity for volcanic ash to incite inflammation in order to better constrain the respiratory hazard posed during future eruptions.

The exact mechanism of how crystalline structures act on inflammasome activation remains elusive. Among others, including ROS involvement, discussed are scavenger receptors such as the macrophage receptor with collagenous structure (MARCO), CD36, or CD204, with MARCO identified as the dominant contributor in the C57BL/6 mouse model (53). However, the contribution of these receptors upon silica crystal uptake with subsequent IL-1β maturation remains controversial, since alveolar macrophages derived from MARCO-null mice show increased IL-1β cytokine release (54). Although scavenger receptor binding or membrane binding of crystalline particles without internalization may also be sufficient for silica-induced IL-1β production (55), lysosomal crystal uptake and procession is required for a potent inflammatory response. It is well accepted that the activation of the NLRP3 inflammasome pathway requires two steps: an initial, for example, NF-κB-mediated, priming step and a second stimulus for NLRP3 assembly with subsequent caspase-1 cleavage. Although we could clearly show that volcanic ash is capable of inducing NLRP3 activation *in vitro*, a link to the initial priming step is still missing. One explanation is provided by the fact that ROS seem to act upstream of NLRP3 activation, involving the NF-κB pathway upon phagocytosis (56, 57), although ROS are capable of performing both activation and inactivation of NF-κB pathways. However, volcanic ash exposure rarely occurs in isolation, for example, there will be concomitant exposure with anthropogenic pollution in populated regions (58), and other phases may initiate signaling. Another priming mechanism was described by Monick et al. (46), involving the ability of ash particles to promote bacterial infection by altered pathogen killing. This, in turn, would lead to an increased microbial burden with enhanced NF-κB signaling (Step 1) *via* pattern recognition receptors and result in a vicious circle.

Chronic inflammation resulting from particle-induced inflammasome activation, for example, in silicosis or asbestosis, is thought to derive from the inability of cells to destroy the ingested material, leading to successive rounds of apoptosis and re-ingestion of the crystalline material (4). Volcanic ash (including MRA5/6/99), however, has proven to be minimally apoptotic and necrotic to macrophages (10, 46), which suggests successful clearance of ash from the lungs. The combination of effective clearance and inflammasome activation may explain previous results from instillation experiments, such as those discussed above by Lee and Richards (11), who observed granuloma in the lymph nodes followed by considerably delayed lung inflammation, but no fibrosis. Prompt transport of ash to the lymphatic system would preclude an immediate and robust response *in situ*, yet inflammasome-initiated signaling may result in the delayed inflammation observed relative to pure crystalline silica. Despite this, in the most comprehensive clinical study to date, no hilar node enlargement was observed in chest X-rays of 37 children on Montserrat who had been exposed to volcanic ash from the Soufrière Hills volcano for 10 years (59). Clinical manifestations of exposure, therefore, are presently unknown.

The disparity in response between pure-phase cristobalite and volcanic ash is attributable, in part, to the heterogeneous nature of both the bulk ash (which is ~15 wt.% cristobalite) and the ash particles themselves (with individual particles often being comprised of various mineral components as seen in Figure 1C). However, the extent of this disparity, as also observed in previous work (10, 12), suggests that the reactivity of volcanic cristobalite itself is suppressed. Crystalline silica reactivity may be contingent on fracturing the surfaces of crystals in equilibrium (60). The high-energy nature of ash generation, through both explosive eruptions and dome-collapse events, ensures that volcanic cristobalite surfaces will be readily and commonly fractured. Nano-scale chemical and structural investigations of surface-exposed cristobalite crystals reveal no evidence for occlusion (8), and the presence of a reactive cristobalite surface has been implicated in the binding of similar proteins by cristobalite-rich volcanic ash and a crystalline silica standard (61). Therefore, intrinsic modifications may act to suppress volcanic cristobalite reactivity beyond the aforementioned diminished surface area-dose, as previously postulated (8, 14). In particular, all volcanic cristobalite is chemically impure, containing up to 3 wt.% aluminum (7, 8); incorporation of structural aluminum at an equivalent dopant concentration has been shown to suppress cristobalite reactivity (15), and may affect the presence of certain surface moieties (i.e., silanols) deemed critical for crystalline silica reactivity (62). Critically, however, volcanic cristobalite appears to be sufficiently reactive to have initiated a response here.

Significant advances in our understanding of the hazard posed by volcanic ash have been made in recent years; however, a serious concern regarding the crystalline silica polymorph cristobalite, the only toxic mineral phase appreciably present in volcanic ash, has persisted. The observations presented herein confirm adherence to the “variable entity” description of crystalline silica, as defined for quartz by Donaldson and Borm (13) and considered for volcanic cristobalite by Horwell et al. (8), whereby the reactive-silica burden for volcanic cristobalite is insufficient to initiate the more immediate and robust response observed with pure crystalline silica. As discussed, previously reported chemical and structural modifications have been hypothesized to alter the pathogenicity of volcanic cristobalite relative to a pure-phase standard (8). We show here, for the first time, that these modifications are insufficient to abrogate reactivity completely. With the potential for volcanic eruptions to impact millions of people, and with so few epidemiological studies having been conducted, mechanistic insight into the potential for ash to cause disease is of immediate public health value. Identification of an established pathway involved in other particle-induced diseases and through which volcanic ash can induce a chronic inflammatory response offers a foundation on which to provide health risk assessments during future volcanic crises.

## Ethics Statement

This study was carried out in accordance with the Ethics Committee of the Ludwig-Maximilians-University of Munich (24.02.2006GP/cp).

## Author Contributions

DED designed the study, performed the particle synthesis and characterization, and drafted the manuscript. PD designed the study, carried out the immunoassays, and drafted the manuscript. CH, PB, UK, MS, and DBD helped conceive the study. All authors read and approved the final manuscript and are accountable for all aspects of the work.

## Conflict of Interest Statement

The authors declare no conflict of interest. The funding bodies had no input into the design of the study, collection, analysis and interpretation of data, or writing of the manuscript.
